# Rural Community Organizations and Mental Health Among Older Adults: Evidence of Dual Economic-Social Pathways in Rural China

**DOI:** 10.3390/healthcare14040525

**Published:** 2026-02-19

**Authors:** Hang Li, Zhibin Li, Huijun Liu

**Affiliations:** 1 Military Preventive Medicine School, Air Force Medical University, Xi’an 710038, China; 2 School of Public Policy and Administration, Xi’an Jiaotong University, Xi’an 710049, China; lizhibin2020@163.com (Z.L.); liuhuij@mail.xjtu.edu.cn (H.L.)

**Keywords:** mental health, older adults, rural community organization, depressive symptoms, life satisfaction

## Abstract

**Highlights:**

**What are the main findings?**

- The establishment of agricultural cooperatives was associated with lower levels of depressive symptoms, while cultural and sports clubs correlated with both reduced depressive symptoms and higher life satisfaction among rural older adults.
- Rural community organizations primarily impact the mental health of older adults through an “economic-social” mechanism.

**What are the implications of the main findings?**

- Policy resources should prioritize community organizations deeply embedded in rural life that combine economic functions with social interaction.
- Supporting these “economic-social” integrated organizations maximizes mental health benefits for older adults.
- Effectively scaling such organizations demands context-specific adaptation to local infrastructure and cultural conditions.

**Abstract:**

**Background/Objectives**: Mental health issues pose a growing public health burden in aging societies, a challenge particularly accentuated in rural China. This study investigated whether the establishment of rural community organizations—specifically volunteer groups, agricultural cooperatives, cultural and sports clubs, senior dance troupes, and senior associations—influences depressive symptoms and life satisfaction among rural older adults. **Methods**: Data were obtained from the Well-being of Elderly Survey in Anhui Province (WESAP) across three waves (2015, 2018, and 2021). The final sample comprised a balanced panel of 511 older adults, providing 1533 observations. A two-way fixed-effects model was employed to analyze the data. **Results**: Empirical results show that the establishment of agricultural cooperatives was associated with lower depressive symptoms. Cultural and sports clubs were associated with reduced depressive symptoms and positively correlated with life satisfaction. Mechanism analysis revealed associations consistent with a dual “economic–social” pathway: establishing agricultural cooperatives was associated with greater economic resilience and stronger social bonds. The establishment of cultural and sports clubs correlated with higher mental well-being, accompanied by increased time spent in social interaction. Notably, volunteer groups and senior associations showed no significant association with mental health outcomes. **Conclusions**: Rural community organizations are critical drivers of mental health equity. Policymakers should prioritize support for organizations that are deeply embedded in rural daily life and integrate economic functions with social interaction to maximize mental health benefits for older adults.

## 1. Introduction

Mental health issues are increasingly emerging as a growing public health challenge within aging societies. Conditions such as depression, anxiety, and loneliness, often precipitated by empty nest syndrome, widowhood, or social disengagement, are widespread among older adults [1]. According to China’s National Report on Mental Health Development, anxiety and depressive symptoms tend to intensify with age, while life satisfaction and psychological resilience often decline. Specifically, the prevalence of depression has been recorded at 12.6%, 16%, and 15.6% across young-old, middle-old, and old-old age groups, respectively. This mental health crisis is particularly acute in rural China, where the average prevalence rate reaches 32.9%, with even higher figures reported among older adults in underdeveloped western provinces [2]. Furthermore, poor mental health can exacerbate physical ailments. For instance, depressive symptoms may worsen health outcomes for seniors with chronic diseases or cognitive impairment, leading to increased suffering, disability, and elevated mortality risks [3]. Consequently, timely mental health intervention is imperative. Guided by active aging initiatives, policymakers have begun promoting social participation among older adults to mitigate this growing crisis. In rural areas, various community organizations have been established, including volunteer groups, agricultural cooperatives, cultural and sports clubs, senior dance troupes, and senior associations, to provide local residents with expanded opportunities for social engagement. This study aims to examine how the establishment of rural community organizations influences depressive symptoms and life satisfaction among older adults, as well as the underlying mechanisms of these effects.

Theoretically, social participation exerts a positive influence on the mental health of older adults. Activity theory is frequently used to explain how social participation or activities relate to mental health in older adults. It posits that older adults who participate in active pursuits in life are more likely to maintain a positive self-concept, contributing to good mental health in later life [4,5,6]. In addition, role accumulation theory also explains the relationship between participation in multiple activities and mental health. The benefits of role accumulation tend to outweigh any stress to which it might give rise, thereby yielding net gratification [7]. These multiple roles can effectively slow the pace of social disengagement by strengthening social networks and social capital.

Empirically, social participation, often measured as a binary variable, has been shown to positively correlate with mental health. Research in this area has examined both whether older adults participate in social activities and the number of activities in which they engage. Several studies have reported a significant negative association between social participation and depressive symptoms [8,9,10]. Social participation appears to play an important protective role against depression in later life [11]. Older adults can expand their social support networks by engaging in active pursuits, which helps reduce loneliness and improve overall well-being [8]. Many studies also have reported a significant positive correlation between social participation and life satisfaction [12,13,14]. Older adults who participate in social activities tend to experience greater happiness and a stronger sense of belonging [15]. Additionally, research has found that older adults who participate in multiple activities have a lower risk of depression and higher life satisfaction. This occurs because participating in diverse social activities provides abundant social resources that help meet varied psychological needs [16].

An extensive amount of research has been conducted on using productive activities and leisure activities as proxies for social participation. However, findings regarding their impact on mental health remain inconsistent. For example, the relationship between paid work and mental health is ambiguous. On one hand, studies have found that older adults who engage in paid work experience lower rates of depressive symptoms and higher life satisfaction. Paid work can provide economic security and social recognition, promoting psychological well-being [11,17,18,19]. On the other hand, some studies have suggested that the relationship between paid work and life satisfaction among older adults is complex and depends on individual attitudes toward work [20]. Additionally, Choi et al. (2021) found that the relationship between paid work and depressive symptoms was not statistically significant after model adjustment [21]. Furthermore, Hong et al. (2009) even pointed out that engaging in paid work in later life may induce depressive symptoms [22].

Volunteer activities are widely recognized as effective for improving the mental health of older adults, though this may primarily apply to urban contexts. Some scholars argued that volunteering strengthens older adults’ social networks and directly improves their subjective well-being [15,18,19,23,24]. In rural areas, older adults often derive greater psychological benefits from volunteering [25]. However, other scholars contended that there is no evidence showing volunteer activities significantly impact depressive symptoms [24]. Considering the resource disparities between urban and rural areas, the protective effect of volunteering appears more pronounced among urban older adults [11].

Mutual-help activities generally have been shown to positively correlate with mental health, but excessive involvement can become a psychological burden. Relevant studies have found that older adults’ donations, informal caregiving, and support to others can foster a sense of self-actualization [11]. Furthermore, mutual-help improves older adults’ cognitive health and indirectly enhances life satisfaction by reducing loneliness [20] and alleviating depressive symptoms [14,18,21]. However, mutual-help activities are not a panacea. For employed older adults, informal caregiving responsibilities can actually increase depressive symptoms [26].

Encouraging physical activity represents a key strategy for improving the mental health of older adults, though its effectiveness may depend on daily activity levels. Numerous studies have found that physical exercise can significantly reduce depressive symptoms [27] and enhance life satisfaction [28,29] among older adults. Toros et al. (2023) emphasized that this effect appears to be associated with greater age and lifetime exercise history, highlighting the value of regular physical exercise in enhancing the quality of life [29]. However, Chinese research has identified an inverted U-shaped relationship between total physical activity dose and depressive symptoms among older adults. When total physical activity doses are extremely high (≥12,000 MET-min/week), the incidence of depressive symptoms actually increases [30]. This suggests that physical activity may be an additional burden for rural older adults engaged in heavy manual labor.

Previous studies have confirmed the relationship between social participation and the mental health of older adults, providing both theoretical and empirical evidence. However, two significant gaps remain. First, these studies often overlook the uniqueness of social participation among rural older adults. The economy and level of social development in rural areas of China are relatively underdeveloped, and the social security system, especially the pension system, is still in its infancy [15]. These structural factors create social participation patterns among rural older adults that differs markedly from that of their urban counterparts. Although some studies have identified urban-rural disparities, few studies have systematically explored the unique patterns of social participation among rural older adults and their underlying psychological mechanisms.

Second, previous studies have overlooked the prerequisites for social participation, such as rural community infrastructure and organizational development. Urban older adults typically have access to abundant community resources and organizational support, enabling them to freely choose from diverse social activities. Conversely, rural older adults are often lack access to community resources, preventing participating in activities that would benefit their physical and mental well-being [11]. This situation underscores the importance of developing community organizations in rural areas, a field that previous studies have not yet systematically explored.

**Figure 1:**
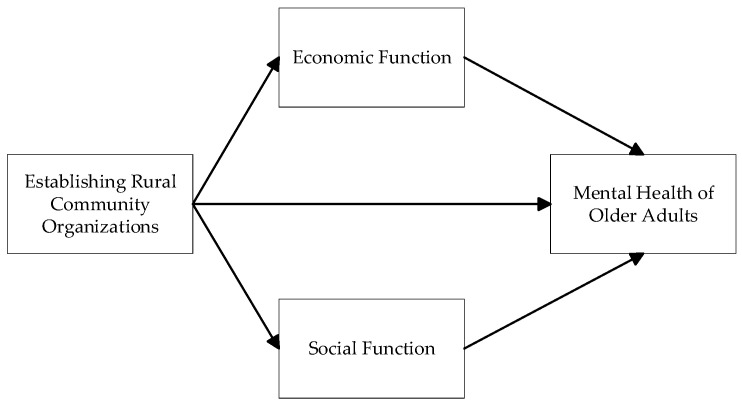
Research Framework of Establishing Rural Community and the Mental Health of Older Adults.

This article focused on the most disadvantaged group, rural older adults, and examined how developing rural community organizations influences the mental health of older adults in these settings. As illustrated in Figure 1, the study first examined the relationship between the establishment of rural community organizations and the mental health of older adults. The organizations encompassed five types, including volunteer groups, agricultural cooperatives, cultural and sports clubs, senior dance troupes, and senior associations. Mental health was measured along two dimensions: depressive symptoms and life satisfaction. This section emphasizes the importance of infrastructure and organizational development. Second, the study investigated the mechanisms through which certain rural community organizations affect older adults’ mental health, highlighting their potential value from both economic and social functional perspectives.

## 2. Materials and Methods

### 2.1. Data and Sample

The present study used data from the survey “Well-being of Elderly Survey in Anhui Province (WESAP),” which comprises both an individual questionnaire and a community-level questionnaire. The survey was conducted every three years from 2001 to 2021 across rural townships in Anhui Province. As of 2024, the old-age dependency ratio in Anhui Province reached 24.17%, surpassing the national average of 22.85% (National Bureau of Statistics). This area was specifically selected for its relatively high density of older adults and high levels of out-migration of working-age adults [12,31,32].

**Figure 2:**
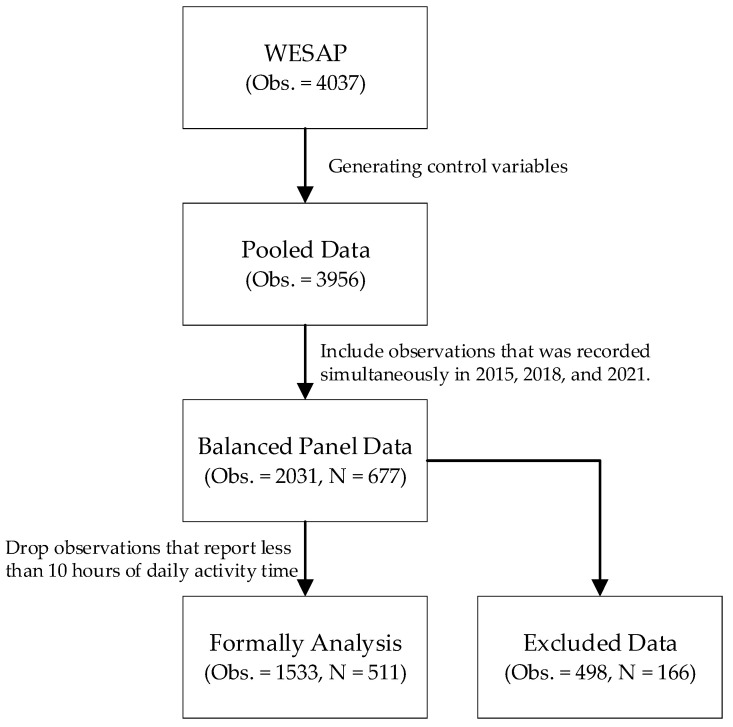
Data Cleaning Process.

A stratified, multi-stage sampling design was employed to conduct a questionnaire survey among permanent residents aged 60 and above in 72 villages across 6 randomly selected townships. The data for this analysis were drawn from three waves, 2015, 2018, and 2021, with sample sizes of 1243, 1234, and 1560, respectively. The data cleaning process is shown in Figure 2. The total number of observations across the three waves was 4037. Some observations were dropped due to missing values when generating control variables, leaving 3965 observations in the pooled dataset. Furthermore, inclusion criteria required participants to have reported their health and socioeconomic status and provided complete time-use diary data across all three waves. A balanced panel was generated with a sample size of 677 and 2031 observations. However, the reported daily activity time varied considerably across participants, potentially leading to measurement errors. The mean daily waking time for older adults was approximately 15.2 h. Some older adults reported more than 12 h of activities, while others reported only 3 to 4 h of activities. To reduce this error, this study removed all observations from the balanced panel that reported activity time of less than 10 h. Samples from 166 individuals (498 observations in total) were excluded. Consequently, the final analytical dataset consisted of 511 older adults, yielding a balanced panel with 1533 observations.

### 2.2. Measures

Dependent Variables. Mental health was operationalized through both positive and negative dimensions. Life satisfaction served as the positive dimension and was measured using the Life Satisfaction Scale [33]. The scale consists of eight items: “(1) Do you feel your life is better than most people? (2) Are you satisfied with your life? (3) Do you find the things you do interesting? (4) Have these past few years been the best days of your life? (5) Would you change your past life if you could do it all over again? (6) Do you find most of what you do to be boring? (7) Do you feel old and find life dull? (8) Do you feel that most of your life has been in line with your own wishes?” Items (5)–(7) are reverse-coded, while all other items are positively phrased. Responses were recorded as “Yes = 1” or “No = 0”. Total scores range from 0 to 8, with higher scores indicating greater life satisfaction. The reliability and validity of this scale have been previously established by Zhang and Silverstein (2022) [34], precluding the need for redundant testing in this study.

Depressive symptoms represented the negative dimension, measured by a revised version of the Center for Epidemiological Studies Depression (CES-D) Scale [35]. The scale comprises nine items: “(1) Do you feel in a good mood? (2) Do you feel lonely? (3) Do you feel deeply sad? (4) Do you think your life is pretty good? (5) Do you feel like you do not want to eat? (6) Do you have trouble in sleeping? (7) Do you feel useless? (8) Do you feel like you have nothing to do? (9) Do you find life to be full of joy?” Items (1), (4), and (9) are positively phrased, while the remaining items are negatively phrased. Responses were recorded as “Often = 2; Sometimes = 1; Never = 0”. Total scores range from 0 to 18, with higher values indicating more severe depressive symptoms. The psychometric properties of this scale have also been validated in prior research [35].

Core Explanatory Variables. The primary explanatory variable is rural community organization. This variable was introduced to examine whether organized social activities in rural areas enhance the mental health of older adults. Five types of rural community organizations, volunteer groups, agricultural cooperatives, cultural and sports clubs, senior dance troupes, and senior associations, were introduced as proxy variables. These organizations facilitate various activities, including social work, productive labor, cultural and recreational pursuits, physical exercise, and rights protection. These variables were derived from the WESAP community questionnaire: “Does your village have the following organizations?” Responses were recorded as binary outcomes “Yes = 1” or “No = 0”. It is worth noting that the core explanatory variable in this study was measured at the community level and exhibited a nested structure. Despite this limitation, the variable remains the most suitable indicator for examining the relationship between the establishment of rural community organizations and the mental health of older adults.

Mediating Variables. For the mechanism analysis, six mediating variables were included: time spent on domestic paid work, time spent on physical exercise, time spent on social interaction, the area of self-cultivated farmland, the total number of friends, and the number of close friends. Time-use variables were captured using a 24 h retrospective diary approach to measure participants’ activities. Total daily hours for each activity were obtained by aggregating individual time periods. The area of self-cultivated farmland was assessed with the question: “What is the current area of farmland you are using?” This variable was recorded as a continuous numerical value. Social network variables (number of friends and close friends) were measured using the following questions: “How many friends do you have whom you interact with at least once a month?” and “How many friends can you confide in about personal matters without reservation?” Responses were recorded as “None = 0; Only one = 1; Two = 2; Three and Four = 3; Five to Eight = 4; Nine and More = 5”.

Control Variables. Following Mao et al. (2023), control variables included (i) demographic characteristics: sex, age, and age-squared; (ii) socioeconomic status: marital status, year of education, and household assets; and (iii) physical health: self-rated health, activities of daily living (ADL), and instrumental activities of daily living (IADL) [8]. Table 1 details the measurement and coding of these control variables. Specifically, Asset of Household was log-transformed to address potential skewness.

Community Variables. The establishment of rural community organizations may be influenced by time-varying village-level factors, such as targeted programs, local economic trends, and leadership. These factors could also affect the mental health of older adults in the community, thus acting as potential confounders. To account for such confounding effects, this study incorporates several time-varying community variables as controls. First, a binary variable was constructed to denote whether the village was officially designated as a national-level poverty village in each survey year (Yes = 1, No = 0). Such villages are eligible for substantial fiscal transfers and development resources, which may not only promote the formation of community organizations but also improve older residents’ well-being. Second, to capture the effect of local economic conditions on organizational development, this study controlled for the village’s gross agricultural product and non-agricultural product in the previous year. Third, since the establishment of community organizations often depends on the discretion and priorities of local leaders, a binary variable indicating whether a village committee election took place in the current year (Yes = 1, No = 0) was also included.

### 2.3. Model

Given that the dependent variables—depressive symptoms and life satisfaction—are continuous and exhibit linear characteristics, this study employed a two-way fixed-effects model. The specific model is as follows:

Y_it_ = β_0_α_it_ + β_1_RuralOrg_it_ + Controls_it_’δ + μ_i_ + η_t_ + ε_it_ (1)

where the subscript *i* denotes older adult *i*, *t* denotes the wave of the survey periods, and *Y_it_* is the dependent variable representing depressive symptoms or life satisfaction of older adult *i* in wave *t*. RuralOrg_it_ is the core explanatory variable, representing whether rural community organization has been established. In addition, Controls_it_ is the matrix of control variables, *μ_i_* and *η_t_* are the fixed effects for the individual and the year, and *ε_it_* is the residual term.

## 3. Results

### 3.1. Descriptive Statistics

Table 2 reports the descriptive statistics for the dependent variables and core explanatory variables. Rows 1 and 2 present the results for depressive symptoms and life satisfaction, respectively. The mean value for depressive symptoms was 5.1182 (SD = 3.5754), suggesting that the overall depressive tendency among older adults in rural Anhui was relatively low. In addition, the mean value for life satisfaction was 5.5445 (SD = 2.1956), indicating a relatively high level of subjective well-being.

Rows 3 through 7 report the descriptive statistics for rural community organizations. Since all these variables are binary, their mean values reflect the proportion of older adults covered by each specific organization. Volunteer groups had a mean value of 0.4207, implying that between 2015 and 2021, 42.07% of the villages where older adults resided had established such groups. Similarly, agricultural cooperatives and cultural and sports clubs were present in 38.85% and 29.14% of the villages, respectively. The sample sizes for senior dance troupes and senior associations were smaller than those of the previous three organizations because these items were first introduced to the questionnaire in 2018, resulting in only two waves of surveyed data. Consequently, senior dance troupes were established in 41.18% of the villages, and senior associations were found in 57.03%.

Rows 8 through 13 display the descriptive statistics for the mediating variables. Within this group, rows 8 to 10 represent time-use variables, indicating the hours older adults allocated to specific activities. Row 8 presents the time spent on domestic paid work, encompassing activities such as farming, livestock care, home businesses, and manual production. The mean value of 2.8928 suggests that older adults in Anhui dedicate approximately three hours per day to domestic paid work. The mean duration for time spent on physical exercise was 0.6834 h, indicating that rural older adults engage in physical activity for approximately 40 min per day. Additionally, with a mean value of 1.4117 for time spent on social interaction, older adults were found to spend roughly 84 min daily on socializing.

Row 11 in Table 2, the area of self-cultivated farmland, represents the land area contracted and cultivated by the older adults’ families. Its mean value was 4.354 mu (Chinese acre). Given that the per capita cultivated land in Anhui Province is approximately 1.17 mu, this descriptive figure is equivalent to the average family-cultivated area for a household of three to four members. Rows 12 and 13 list the number of friends and the number of close friends, respectively. The mean number of friends was 1.5955, indicating an average of one or two friends within the same village. The mean number of close friends (1.4181) was slightly lower than the total number of friends, also averaging between one and two individuals.

Rows 14 through 22 report the descriptive statistics for the demographic characteristics, socioeconomic status, and physical health indicators of older adults. Specifically, the mean value for sex was 0.5108, indicating that 51.08% of the observations were male. The age distribution ranged from 60 to 97 years, with a mean age of 71.46 years. The mean score for self-rated health was 2.5134 (SD = 0.9974), suggesting that most older adults perceived their health as being between “moderate” (=2) and “good” (=3). The mean scores for ADL and IADL were 76.3079 and 14.5829, respectively, reflecting relatively high levels of intrinsic capacity and functional ability. The mean for marital status was 0.7097, indicating that the majority of respondents were currently married or had marital experience. The average years of education was 2.6523, implying that most older adults had attended school for two to three years, typically reaching an elementary education level. Finally, the mean household assets value was 13,328.26; the log-transformed form of this variable will be used in the regression analysis.

The rest of Table 2 reports the descriptive statistics for community variables. The mean value of national poverty village was 0.1367, indicating that 13.67% of the observations corresponded to villages designated as national poverty villages. The average agricultural gross production and non-agricultural gross production of these villages were 14,527.52 and 2577.67, respectively. In 6.41% of observations, village committee elections were held during the survey year.

### 3.2. Establishment of Rural Community Organizations and Mental Health of Older Adults

Table 3 presents the estimated results of rural community organization establishment on depressive symptoms among older adults. Columns (1)–(5) used OLS estimators, and all models incorporate control variables along with fixed effects for the individual and the year. In Table 3, the only statistically significant coefficients observed were for agricultural cooperatives and cultural and sports clubs. The coefficient for agricultural cooperatives was −0.4656 (*p* < 0.05), indicating that a one-standard-deviation increase in this variable is associated with a 6.16% reduction in the standard deviation of depressive symptoms (= −0.4655 × 0.4733/3.5753). The coefficient of cultural and sports clubs was −0.7736 and significant at the 1% level, indicating that a one-standard-deviation increase corresponds to a 9.84% (= −0.7736 × 0.4546/3.5754) decrease in the standard deviation of depressive symptoms.

Table 4 reports the results of rural community organization establishment on life satisfaction among older adults. Across all models, only the coefficient for cultural and sports clubs reached statistical significance at the 10% level. The coefficient of 0.3141 implies that a one-standard-deviation increase in cultural and sports clubs is associated with a 6.50% (=0.3141 × 0.4546/2.1956) increase in the standard deviation of life satisfaction. These findings suggest that cultural and sports clubs hold significant potential as interventions for improving older adults’ mental health in terms of both depressive symptoms and life satisfaction.

The establishment of rural community organizations may be influenced by village-level confounding factors. Among these, most confounding factors are time-invariant, including the geographical location and demographic structure of villages. This study employed a high-dimensional fixed-effects model, incorporating both individual, year and village fixed effects into the model to control for time-invariant confounding. Village fixed effects were not included because older adults rarely migrate across villages in this setting; consequently, individual fixed effects fully absorb village-level variation, leading to perfect multicollinearity. Therefore, village fixed effects were excluded from the final models to maintain model identification.

To identify time-varying confounding, Table 5, Table 6 and Table 7 present models that incorporate four community variables. These variables included national poverty village, gross agricultural product, gross non-agricultural product, and village committee elections. Table 5 shows the coefficients of agricultural cooperatives after the stepwise incorporation of community variables. The coefficients ranged from −0.4736 to −0.4202 and were significant at the 10% to 5% level. These results are similar to the coefficient in Table 3 (−0.4656), indicating that adding community variables does not affect the core conclusions of this study. In addition, none of the community variables showed a significant association with depressive symptoms among older adults.

Table 6 shows the coefficients of cultural and sports clubs after the stepwise incorporation of community variables. Similar to Table 5, the coefficients ranged from −0.8070 to −0.7657 and were significant at the 1% level. These results are similar to the coefficient in Table 3 (−0.7736), indicating that adding community variables does not affect the conclusions regarding the mental health effects of cultural and sports clubs. In addition, none of the community variables showed a significant association with depressive symptoms among older adults.

Table 7 reports the association between establishing cultural and sports clubs and life satisfaction of older adults. The coefficients ranged from 0.3016 to 0.3173 and were significant at the 10% level. These results are similar to the coefficient in Table 4 (0.3141), indicating that adding community variables does not affect the conclusions regarding the effect of cultural and sports clubs on life satisfaction. additionally, the coefficient of national poverty village was −0.6910 and significant at the 5% level. Even after incorporating other community variables, the coefficient remained −0.7059 and significant at the 10% level. These results may suggest that older adults living in villages designated as poverty villages by the government experience lower levels of life satisfaction.

### 3.3. Analysis of the Mechanism of Rural Community Organizations on the Mental Health of Older Adults

The establishment of agricultural cooperatives was correlated with depressive symptoms. The underlying mechanisms likely involve both economic and social dimensions. From an economic perspective, the mutual aid provided by agricultural cooperatives assists older adults in farming, thereby alleviating the burden of heavy physical labor through the use of agricultural machinery. Rather than flowing into leisure activities, the time freed up from farming continues to be allocated to productive activities. Some older adults may continue to spend time on farming, which is reflected in renting more farmland. Others may spend time on domestic paid work, such as livestock care or handicraft production. Regardless of the type of productive activity engaged in, the establishment of agricultural cooperatives is likely to raise the income of rural older adults and strengthen their economic resilience. To explore this, two mediating variables, time spent on domestic paid work and area of self-cultivated farmland, were introduced into the analysis. The support provided by agricultural cooperatives may lead to an increase in both the area of self-cultivated farmland and the associated labor hours.

Results for this mechanism are presented in Columns (1) and (2) of Table 8. In Column (1), the coefficient was 0.4115 (*p* < 0.10), indicating that a one-standard-deviation increase in agricultural cooperatives is associated with a 5.80% (=−0.4115 × 0.4734/3.3566) increase in the standard deviation of the time spent on domestic paid work. In other words, the establishment of these cooperatives is associated with a 0.4115 h (approximately 24 min) increase in the daily domestic paid work performed by older adults. In Column (2), the coefficient of 0.5643 (*p* < 0.10) indicates that a one-standard-deviation increase in agricultural cooperatives corresponds to a 5.27% (=0.5643 × 0.4733/5.0651) increase in the standard deviation of the area of self-cultivated farmland. This suggests that the establishment of these cooperatives is linked to an increase of 0.5643 mu in the area of self-cultivated farmland.

From a social perspective, agricultural cooperatives may indirectly foster social interaction among older adults by promoting mutual aid, as reflected in an increase in the number of friends. Accordingly, the number of friends and the number of close friends were introduced as mediating variables. The establishment of agricultural cooperatives is expected to increase both the number of friends and close friends. Columns (3) and (4) of Table 8 report the results for this social mechanism. In Column (3), the coefficient was 0.2275 (*p* < 0.05), suggesting that a one-standard-deviation increase in agricultural cooperatives is associated with a 7.36% (=0.2275 × 0.4733/1.4630) increase in the standard deviation of the number of friends. In Column (4), the coefficient for the number of close friends was 0.1632 (*p* < 0.10), indicating that a one-standard-deviation increase in cooperatives is associated with a 5.66% (=0.1632 × 0.4733/1.3656) increase in the standard deviation of close friends.

The establishment of cultural and sports clubs was associated with decreased depressive symptoms and increased life satisfaction. One potential explanation is that these clubs foster social interaction among older adults. Therefore, the time spent on social interaction was included as a mediating variable in the analysis. Columns (1) and (2) of Table 9 report the results. In Column (1), although cultural and sports clubs were positively correlated with time spent on physical exercise, the coefficient did not reach statistical significance. In Column (2), the coefficient was 0.2861 (*p* < 0.10), indicating that a one-standard-deviation increase in cultural and sports clubs is associated with a 6.55% (=0.2861 × 0.4546/1.9859) increase in the standard deviation of time spent on social interaction. In practical terms, the establishment of these clubs is associated with an average increase of 0.2861 h (approximately 17 min) in the time older adults dedicate to social interaction.

## 4. Discussion

Rural community organizations serve as critical conduits for social engagement and pivotal drivers in advancing active aging and mental health equity. This study examined the relationship between the establishment of rural community organizations and the mental health of older adults. Empirical results revealed a significant association between the establishment of agricultural cooperatives and lower levels of depressive symptoms among older adults. Furthermore, the establishment of cultural and sports clubs was associated with decreased depressive symptoms and increased life satisfaction. These findings suggest that fostering rural community organizations constitutes a valuable strategy for enhancing geriatric mental health in this region.

Our study reveals a dual “economic-social” mechanism through which rural community organizations influence the mental health of older adults. This finding provides empirical support for activity theory, which posits that creating an active environment in social life helps older adults maintain a positive self-concept and psychological well-being. On the one hand, agricultural cooperatives and cultural and sports clubs fulfill essential social functions, which directly align with role accumulation theory. The establishment of these organizations was positively correlated with the number of friends and the time spent on social interaction, demonstrating their potential to strengthen social bonds. On the other hand, agricultural cooperatives perform a distinct economic function. Establishing agricultural cooperatives was positively correlated with the time spent on domestic paid work and the area of self-cultivated farmland. These findings highlight the importance of agricultural mutual aid in improving the economic resilience of households and promoting the mental health of rural older adults.

This study contributes to several strands of the existing literature. First, its primary marginal contribution lies in uncovering the dual “economic-social” mechanism through which rural community organizations influence the mental health of older adults. While Zhou et al. (2023) argued that “self-employment” indirectly improves mental health through income growth and self-actualization [16], our findings offer a divergent perspective. In rural settings, where labor hours for older adults are substantially higher than those of their urban counterparts [37], merely extending working hours may increase psychological burdens rather than improve well-being. In contrast, rural community organizations do not rely on simple labor extension; instead, they alleviate the pressure of high-intensity labor through agricultural mutual aid, provide production support, and enhance family economic resilience, thereby promoting mental health.

Second, this study identifies that not all types of rural community organizations are equally effective, offering a nuanced boundary condition for activity theory. Notably, the establishment of volunteer groups, senior dance troupes, and senior associations showed no significant association with mental health. This suggests that activities centered on volunteering, mutual help, or general physical exercise may offer limited mental health benefits within the current rural Chinese context. While Guo et al. (2018) attributed such phenomena to structural inequalities in resource distribution [11], our results suggest deeper constraints. A plausible explanation is that excessive working hours among rural older adults severely deplete the time and energy required for leisure or productive social participation, thereby attenuating the potential health-promoting effects of these organizations.

The policy implications of this research are twofold. First, it is essential to develop rural community organizations that align with the established habits and core needs of the rural older adults. Policy resources should prioritize organizations that are deeply embedded in daily rural life and can effectively reduce labor burdens or provide meaningful socialization. Second, older adults should be encouraged to participate in community organizations, particularly those that integrate economic functions with social interaction. Such integrated “economic-social” activities have been shown to enhance economic resilience and social interaction, thereby promoting mental well-being. Third, tailoring organizational support to local economic and cultural contexts is essential for broader applicability. Policymakers should consider regional heterogeneity when scaling up interventions through rural community organizations. While our findings from central Anhui highlight the mental health benefits of economically embedded community organizations, their effectiveness may vary across rural China. In less developed western villages with weaker infrastructure, the economic function may be constrained; in southern areas with strong kinship networks, the social role may be partially substituted.

This study has two primary limitations. First, the measurement of rural community organizations is not exhaustive. We used binary variables to indicate the presence of specific organizations. While this approach ensures operational feasibility, it fails to capture critical dimensions such as activity frequency, specific content, or participation rates, potentially introducing endogeneity related to measurement error. Second, some mediating variables may be subject to measurement error. Time-use variables were based on self-reported recall, which can be affected by age-related declines in cognitive function and memory of older adults. For instance, respondents might forget certain activities they engaged in, or become fatigued during the lengthy questionnaire and consequently omit reporting some activities. This could lead to an underestimation of the health benefits associated with the establishment of rural community organizations.

## 5. Conclusions

This study contributes to the literature on active aging and rural mental health. Our findings delineate the pathways through which rural community organizations enhance mental health via a dual “economic-social” mechanism: establishing agricultural cooperatives was associated with greater economic resilience and stronger social bonds. The establishment of cultural and sports clubs was correlated with higher mental well-being, accompanied by increased time spent on social interaction. Given the context of rapid population aging, policymakers should prioritize support for community organizations that are deeply rooted in rural life and possess both economic and social attributes to maximize mental health benefits for older adults.

## Figures and Tables

**Table 1:** Coding of Control Variables.

Variables	Coding
*Sex*	Sex = 1 (Male); = 0 (Female)
*Age*	Age of older adult
*Age_sqr*	Squared age of older adult
*Sr_health*	Self-rated health = 1 (Bad); = 2 (Poor); = 3 (Good); = 4 (Excellent)
*ADL*	Generated by Barthel ADL Index (BI)
*IADL*	Generated by Functional Activities Questionnaire (FAQ)
*Marital Status*	Marital status = 1 (Married); = 0 (Not Married)
*Year of Education*	Year of schooling for older adult
*Asset of Household*	Log-transformed asset of household (CNY)

**Table 2:** Descriptive Statistics.

Variables	Categories	Obs.	Mean	S.D.	Min	Max
*Depressive Symptoms*	Dependent Variables	1515	5.1182	3.5754	0	17
*Life Satisfaction*		1493	5.5445	2.1956	0	8
*Volunteer Groups*	Core Explanatory Variables	1338	0.4207	0.4938	0	1
*Agricultural Cooperatives*		1338	0.3385	0.4733	0	1
*Cultural and Sports Clubs*		1338	0.2914	0.4546	0	1
*Senior Dance Troupes*		896	0.4118	0.4924	0	1
*Senior Associations*		896	0.5703	0.4953	0	1
*Time Spent on Domestic Paid Work*	Mediating Variables	1533	2.8928	3.3294	0	12.5
*Time Spent on Physical Exercise*		1533	0.6834	1.2502	0	6.3256
*Time Spent on Social Interaction*		1533	1.4117	1.9859	0	7.9325
*Area of Self-cultivated Farmland*		1533	4.354	5.0651	0	30
*Number of Friends*		1533	1.5955	1.463	0	5
*Number of Close Friends*		1533	1.4181	1.3656	0	5
*Sex*	Control Variables	1533	0.5108	0.5004	0	1
*Age*		1533	71.469	7.2259	60	97
*Age_sqr*		1533	5160	1075.87	3481	9409
*Sr_health*		1529	2.5134	0.9974	1	4
*ADL*		1533	76.3079	9.563	10	80
*IADL*		1520	14.5829	3.1021	0	18
*Marital Status*		1533	0.7097	0.454	0	1
*Year of Education*		1533	2.6523	3.4032	0	16
*Asset of Household*		1533	13,328.62	36,728.6	0	700,000
*National Poverty Village*	Community Variables	1338	0.1367	0.3437	0	1
*Gross Agricultural Product*		1338	14,527.52	92,331.28	0	790,000
*Gross Non-Agricultural Product*		1338	2577.67	4596.037	0	30,631.1
*Village Committee Election*		1527	0.0641	0.2451	0	1

**Table 3:** Relationship between Establishing Rural Community Organizations and Depression Symptoms among Older Adults.

	Depressive Symptoms				
	(1)	(2)	(3)	(4)	(5)
*Volunteer Groups*	0.5779				
	(0.4929)				
*Agricultural Cooperatives*		−0.4656 **			
		(0.1813)			
*Cultural and Sports Clubs*			−0.7736 ***		
			(0.2748)		
*Senior Dance Troupes*				−0.0456	
				(0.3085)	
*Senior Associations*					−0.2727
					(0.3025)
Controls	Yes	Yes	Yes	Yes	Yes
Individual fixed effects	Yes	Yes	Yes	Yes	Yes
Year fixed effects	Yes	Yes	Yes	Yes	Yes
Obs.	1321	1321	1321	876	876
F-stat.	18.7018	29.6223	12.4185	6.5295	6.6859
Within R-sq	0.1238	0.1235	0.1271	0.1548	0.1563

Notes: *** *p* < 0.01, ** *p* < 0.05. All models used clustered standard errors. As the majority of older adults only participate in activities within their own village, the influence of rural community organizations is largely confined to the village level. Therefore, the standard errors were clustered by village. This table is a reduced version. The full table can be found in the Supplementary Materials (Table S1).

**Table 4:** Relationship between Establishing Rural Community Organizations and Life Satisfaction among Older Adults.

	Life Satisfaction				
	(1)	(2)	(3)	(4)	(5)
*Volunteer Groups*	−0.2222				
	(0.1764)				
*Agricultural Cooperatives*		0.1394			
		(0.1362)			
*Cultural and Sports Clubs*			0.3141 *		
			(0.1693)		
*Senior Dance Troupes*				−0.0548	
				(0.3361)	
*Senior Associations*					−0.2144
					(0.2993)
Controls	Yes	Yes	Yes	Yes	Yes
Individual fixed effects	Yes	Yes	Yes	Yes	Yes
Year fixed effects	Yes	Yes	Yes	Yes	Yes
Obs.	1298	1298	1298	856	856
F-stat.	17.0333	32.6071	11.0634	36.132	29.2131
Within R-sq	0.1108	0.1100	0.1124	0.1424	0.1448

Notes: This table is a reduced version. The full table can be found in the Supplementary Materials (Table S2). * *p* < 0.1

**Table 5:** Relationship between Establishing Agricultural Cooperatives and Depressive Symptoms with Community Variables.

	Depressive Symptoms			
	(1)	(2)	(3)	(4)
*Agricultural Cooperatives*	−0.4358 *	−0.4736 **	−0.4357 **	−0.4202 **
	(0.2034)	(0.1725)	(0.1711)	(0.1814)
*National Poverty Village*	0.4767			0.4291
	(0.6673)			(0.6883)
*Gross Agricultural Product*		−6.05 × 10^−7^		−6.04 × 10^−7^
		(1.15 × 10^−6^)		(1.13 × 10^−6^)
*Gross Non-Agricultural Product*		7.24 × 10^−6^		4.28 × 10^−6^
		(3.21 × 10^−5^)		(2.95 × 10^−5^)
*Village Committee Election*			0.2411	0.2084
			(0.4680)	(0.4702)
Controls	Yes	Yes	Yes	Yes
Individual fixed effects	Yes	Yes	Yes	Yes
Year fixed effects	Yes	Yes	Yes	Yes
Obs.	1321	1321	1321	1321
F-stat.	12.19	11.16	12.49	9.52
Within R-sq	0.1243	0.1239	0.1239	0.125

Notes: This table is a reduced version. The full table can be found in the Supplementary Materials (Table S3). ** *p* < 0.05, * *p* < 0.1.

**Table 6:** Relationship between Establishing Cultural and Sports Clubs and Depressive Symptoms with Community Variables.

	Depressive Symptoms			
	(1)	(2)	(3)	(4)
*Cultural and Sports Clubs*	−0.7730 ***	−0.8070 ***	−0.7657 ***	−0.8019 ***
	(0.2781)	(0.2838)	(0.2794)	(0.2804)
*National Poverty Village*	0.6299			0.5783
	(0.5387)			(0.5550)
*Gross Agricultural Product*		−9.51 × 10^−7^		−9.66 × 10^−7^
		(8.88 × 10^−7^)		(8.90 × 10^−7^)
*Gross Non-Agricultural Product*		3.89 × 10^−6^		1.83 × 10^−7^
		(1.77 × 10^−5^)		(1.77 × 10^−5^)
*Village Committee Election*			0.3776	0.3192
			(0.3908)	(0.3916)
Controls	Yes	Yes	Yes	Yes
Individual fixed effects	Yes	Yes	Yes	Yes
Year fixed effects	Yes	Yes	Yes	Yes
Obs.	1321	1321	1321	1321
F-stat.	10.78	9.75	10.74	8.38
Within R-sq	0.1286	0.1279	0.1281	0.1302

Notes: This table is a reduced version. The full table can be found in the Supplementary Materials (Table S4). *** *p* < 0.01.

**Table 7:** Relationship between Establishing Cultural and Sports Clubs and Life Satisfaction with Community Variables.

	Life Satisfaction			
	(1)	(2)	(3)	(4)
*Cultural and Sports Clubs*	0.3124 *	0.3016 *	0.3173 *	0.3087 *
	(0.1712)	(0.1750)	(0.1730)	(0.1726)
*National Poverty Village*	−0.6910 **			−0.7059 *
	(0.3496)			(0.3625)
*Gross Agricultural Product*		−1.80 × 10^−7^		−1.55 × 10^−7^
		(5.64 × 10^−7^)		(5.65 × 10^−7^)
*Gross Non-Agricultural Product*		−1.10 × 10^−5^		−4.82 × 10^−6^
		(1.42 × 10^−5^)		(1.47 × 10^−5^)
*Village Committee Election*			0.1518	0.2167
			(0.2279)	(0.2293)
Controls	Yes	Yes	Yes	Yes
Individual fixed effects	Yes	Yes	Yes	Yes
Year fixed effects	Yes	Yes	Yes	Yes
Obs.	1298	1298	1298	1298
F-stat.	9.76	8.72	9.87	7.85
Within R-sq	0.1172	0.1132	0.1128	0.1183

Notes: This table is a reduced version. The full table can be found in the Supplementary Materials (Table S5). ** *p* < 0.05, * *p* < 0.1.

**Table 8:** Mechanism of Agricultural Cooperatives on Depression Symptoms among Older Adults.

	Time Spent on Domestic Paid Work	Area of Self-Cultivated Farmland	Number of Friends	Number of Close Friends
	**(1)**	**(2)**	**(3)**	**(4)**
*Agricultural Cooperatives*	0.4115 *	0.5643 *	0.2275 **	0.1632 *
	(0.2118)	(0.3205)	(0.0913)	(0.0887)
	[0.1988]			
Controls	Yes	Yes	Yes	Yes
Fixed effects	Yes	Yes	Yes	Yes
Obs.	1338	1338	1338	1338
F-stat.	1.4176	1.5	2.55	2.83
Wald chi-sq	49.55			
Within R-sq	0.0122	0.0142	0.0229	0.0249

Notes: This table presents the results for time-use variables. It is worth noting that older adults’ participation in a given social activity depends on their lifestyle, so the time they spend on that activity can vary substantially. Measuring time-use variables reduces the sample size for certain social activities. Meanwhile, clustered standard errors were used in our model, leading to a lower degree of freedom and validity. To address this, we followed Abadie et al. (2022) and report two types of standard errors for the time-use variables [36]. The first type, clustered standard errors, is shown in parentheses. The second type, standard errors obtained from 1000 bootstrap replications, is shown in square brackets. Additionally, using either type of standard error does not alter the substantive conclusions of this article. This table is a reduced version. The full table can be found in the Supplementary Materials (Table S6). ** *p* < 0.05, * *p* < 0.1.

**Table 9:** Mechanism of Cultural and Sports Clubs on Mental Health among Older Adults.

	Time Spent on Physical Exercise	Time Spent on Social Interaction
	(1)	(2)
*Cultural and Sports Clubs*	0.0465	0.2861 *
	(0.1505)	(0.1478)
	[0.1034]	[0.1578]
Controls	Yes	Yes
Fixed effects	Yes	Yes
Obs.	1338	1338
F-stat.	10.39	2.15
Wald chi-sq	37.66	19.83
Within R-sq	0.012	0.0203

Notes: The clustered standard errors were reported in parentheses. The standard errors calculated by 1000 Bootstrap-based replications are reported in square brackets. Additionally, using either type of standard error does not alter the substantive conclusions of this article. This table is a reduced version. The full table can be found in the Supplementary Materials (Table S7). * *p* < 0.1.

## Data Availability

The raw data supporting the conclusions of this article will be made available by the authors on request.

## Supplementary Materials

The following supporting information can be downloaded at: https://www.mdpi.com/article/10.3390/healthcare14040525/s1. The full tables of regression results are attached in the Supplementary File. Table S1 (Full table of Table 3 in manuscript); Table S2 (Full table of Table 4 in manuscript); Table S3 (Full table of Table 5 in manuscript); Table S4 (Full table of Table 6 in manuscript); Table S5 (Full table of Table 7 in manuscript); Table S6 (Full table of Table 8 in manuscript); Table S7 (Full table of Table 9 in manuscript).
