# Supplementary material for: Rural Community Organizations and Mental Health Among Older Adults: Evidence of Dual Economic-Social Pathways in Rural China
Source: Healthcare (Basel). 2026 Feb 19;14(4):525. doi: 10.3390/healthcare14040525 (PMC12940825; doi:10.3390/healthcare14040525)
Supplement: Supplementary file 1 [file healthcare-14-00525-s001.zip › healthcare-4137076-supplementary.pdf]

Supplementary File

This document is used to present the full tables of the main text.

Table S1 (Full table of Table 3 in manuscript)

	(1)	(2)	(3)	(4)	(5)
Volunteer Groups	0.5779				
	(0.4929)				
Agricultural Cooperatives		-0.4656**			
		(0.1813)			
Cultural and Sports Clubs			-0.7736***		
			(0.2748)		
Senior Dance Troupe				-0.0456	
				(0.3085)	
Senior Associations					-0.2727
					(0.3025)
Sex	0	0	0	0	0
	(.)	(.)	(.)	(.)	(.)
Age	-0.0558	-0.0778	-0.0533	1.0516	1.0373
	(0.6931)	(0.6715)	(0.4511)	(0.8221)	(0.8203)
Age_sqr	0.0018	0.002	0.0019	-0.0071	-0.0069
	(0.0035)	(0.0035)	(0.0025)	(0.0047)	(0.0047)
Sr_health	-0.8119***	-0.8033***	-0.7961***	-0.8240***	-0.8227***
	(0.2053)	(0.2000)	(0.1270)	(0.1721)	(0.1705)
ADL	-0.0128	-0.0142	-0.0149	-0.0084	-0.0086
	(0.0170)	(0.0163)	(0.0161)	(0.0202)	(0.0203)
IADL	0.2073***	0.2171***	0.2141***	0.3105***	0.3071***
	(0.0522)	(0.0531)	(0.0556)	(0.0812)	(0.0810)
Marital Status	-0.9419**	-0.8559*	-0.8904**	-0.6419	-0.6668
	(0.4141)	(0.4167)	(0.4258)	(0.6841)	(0.6813)
Year of Education	-0.0004	0.0055	0.0078	-0.0863	-0.0823
	(0.0503)	(0.0525)	(0.0471)	(0.0658)	(0.0658)
Asset of Household	0	0	0	0	0
	(0.0000)	(0.0000)	(0.0000)	(0.0000)	(0.0000)
_cons	2.6611	3.568	2.3226	-31.4836	-31.14
	(32.6096)	(31.5167)	(21.1185)	(37.4052)	(37.2904)
N	1321	1321	1321	876	876
F	18.7018	29.6223	12.4185	6.5295	6.6859
r2	0.1238	0.1235	0.1271	0.1548	0.1563

Notes: ***p < 0.01, **p < 0.05, *p < 0.1. The same as below.

Table S2 (Full table of Table 4 in manuscript)

	(1)	(2)	(3)	(4)	(5)
Volunteer Groups	-0.2222				
	(0.1764)				
Agricultural Cooperatives		0.1394			
		(0.1362)			
Cultural and Sports Clubs			0.3141*		
			(0.1693)		
Senior Dance Troupe				-0.0548	
				(0.3361)	
Senior Associations					-0.2144
					(0.2993)
Sex	0	0	0	0	0
	(.)	(.)	(.)	(.)	(.)
Age	0.0937	0.109	0.0963	0.3959	0.3748
	(0.2957)	(0.2952)	(0.2918)	(0.6697)	(0.6551)
Age_sqr	-0.0011	-0.0012	-0.0012	-0.0018	-0.0016
	(0.0013)	(0.0013)	(0.0017)	(0.0039)	(0.0039)
Sr_health	0.5580***	0.5529***	0.5511***	0.6057***	0.6074***
	(0.1035)	(0.1052)	(0.0761)	(0.1093)	(0.1049)
ADL	-0.0042	-0.0036	-0.0031	-0.0082	-0.0083
	(0.0141)	(0.0145)	(0.0107)	(0.0166)	(0.0154)
IADL	-0.1283**	-0.1312***	-0.1300***	-0.1324**	-0.1356**
	(0.0418)	(0.0416)	(0.0364)	(0.0516)	(0.0514)
Marital Status	0.3304	0.3015	0.3107	0.2119	0.1931
	(0.2228)	(0.2316)	(0.3124)	(0.3350)	(0.3768)
Year of Education	-0.0125	-0.0139	-0.015	0.0126	0.0155
	(0.0272)	(0.0258)	(0.0277)	(0.0470)	(0.0483)
Asset of Household	0	0	0	0.0000**	0.0000*
	(0.0000)	(0.0000)	(0.0000)	(0.0000)	(0.0000)
_cons	3.9384	3.1249	3.7721	-14.4048	-13.6685
	(15.5303)	(15.4829)	(13.2775)	(29.1204)	(27.8149)
N	1298	1298	1298	856	856
F	17.0333	32.6071	11.0634	36.132	29.2131
r2	0.1108	0.1100	0.1124	0.1424	0.1448

Table S3 (Full table of Table 5 in manuscript)

	[1]	[2]	[3]	[4]
Agricultural Cooperatives	-0.4358*	-0.4736**	-0.4357**	-0.4202**
	[0.2034]	[0.1725]	[0.1711]	[0.1814]
National Poverty Village	0.4767			0.4291
	[0.6673]			[0.6883]
Gross Agricultural Product		-6.05E-07		-6.04E-07
		[1.15e-06]		[1.19e-06]
Gross Non-Agricultural Product		7.24E-06		4.28E-06
		[0.0000321]		[0.000024]
Village Committee Election			0.2411	0.2084
			[0.4680]	[0.4702]
Sex	0	0	0	0
	[.]	[.]	[.]	[.]
Age	-0.1026	-0.0798	-0.0647	-0.091
	[0.6884]	[0.6704]	[0.6797]	[0.6964]
Age_sqr	0.0021	0.002	0.0019	0.002
	[0.0036]	[0.0035]	[0.0035]	[0.0036]
Sr_health	-0.7978***	-0.8026***	-0.8061***	-0.8003***
	[0.1971]	[0.2004]	[0.2000]	[0.1975]
ADL	-0.0142	-0.0137	-0.0133	-0.0131
	[0.0163]	[0.0167]	[0.0163]	[0.0164]
IADL	0.2169***	0.2180***	0.2198***	0.2197***
	[0.0522]	[0.0536]	[0.0542]	[0.0528]
Marital Status	-0.8650*	-0.8583*	-0.8595*	-0.8736*
	[0.4245]	[0.4267]	[0.4172]	[0.4385]
Year of Education	0.0068	0.0055	0.0057	0.0069
	[0.0528]	[0.0532]	[0.0527]	[0.0535]
Asset of Household	-4.59E-07	-6.16E-07	-6.00E-07	-5.39E-07
	[3.29e-06]	[3.37e-06]	[3.28e-06]	[3.86e-06]
_cons	4.5996	3.7267	2.9699	4.1828
	[32.1479]	[31.2339]	[31.8979]	[32.2902]
N	1321	1321	1321	1321
F	12.19	11.16	12.49	9.52

Table S4 (Full table of Table 6 in manuscript)

	[1]	[2]	[3]	[4]
Agricultural Cooperatives	-0.7730***	-0.8070***	-0.7657***	-0.8019***
	[0.2781]	[0.2838]	[0.2794]	[0.2804]
National Poverty Village	0.6299			0.5783
	[0.5387]			[0.5550]
Gross Agricultural Product		-9.51E-07		-9.66E-07
		[8.88e-07]		[8.90e-07]
Gross Non-Agricultural Product		3.89E-06		1.83E-07
		[1.77E-05]		[1.77E-05]
Village Committee Election			0.3776	0.3192
			[0.3908]	[0.3916]
Sex	0	0	0	0
	[.]	[.]	[.]	[.]
Age	-0.0856	-0.0561	-0.0324	-0.0685
	[0.4641]	[0.4626]	[0.4644]	[0.4679]
Age_sqr	0.0021	0.0019	0.0018	0.0019
	[0.0026]	[0.0026]	[0.0026]	[0.0026]
Sr_health	-0.7902***	-0.7956***	-0.8020***	-0.7955***
	[0.1304]	[0.1304]	[0.1309]	[0.1316]
ADL	-0.015	-0.0144	-0.0136	-0.0135
	[0.0164]	[0.0165]	[0.0164]	[0.0165]
IADL	0.2142***	0.2145***	0.2187***	0.2180***
	[0.0569]	[0.0571]	[0.0567]	[0.0568]
Marital Status	-0.8980**	-0.9048**	-0.8910**	-0.9174**
	[0.4392]	[0.4383]	[0.4382]	[0.4450]
Year of Education	0.0096	0.0083	0.0082	0.0105
	[0.0485]	[0.0483]	[0.0485]	[0.0487]
Asset of Household	-1.64E-06	-1.95E-06	-1.82E-06	-1.76E-06
	[3.72e-06]	[3.76e-06]	[3.74e-06]	[3.75e-06]
_cons	3.6643	2.6263	1.3746	3.1144
	[21.6938]	[21.6591]	[21.8108]	[21.9315]
N	1321	1321	1321	1321
F	10.7793	9.7501	10.7357	8.3807

Table S5 (Full table of Table 7 in manuscript)

	[1]	[2]	[3]	[4]
Agricultural Cooperatives	0.3124*	0.3016*	0.3173*	0.3087*
	[0.1712]	[0.1750]	[0.1730]	[0.1726]
National Poverty Village	-0.6910**			-0.7059*
	[0.3496]			[0.3625]
Gross Agricultural Product		-1.8E-07		-1.55E-07
		[5.64e-07]		[5.65e-07]
Gross Non-Agricultural Product		-0.000011		-4.82E-06
		[1.42E-05]		[1.47E-05]
Village Committee Election			0.1518	0.2167
			[0.2279]	[0.2293]
Sex	0	0	0	0
	[.]	[.]	[.]	[.]
Age	0.1352	0.0927	0.1037	0.1447
	[0.3005]	[0.2977]	[0.2990]	[0.3005]
Age_sqr	-0.0014	-0.0012	-0.0012	-0.0015
	[0.0017]	[0.0017]	[0.0017]	[0.0017]
Sr_health	0.5448***	0.5502***	0.5484***	0.5404***
	[0.0777]	[0.0782]	[0.0784]	[0.0783]
ADL	-0.0032	-0.0034	-0.0026	-0.0024
	[0.0109]	[0.0109]	[0.0110]	[0.0110]
IADL	-0.1304***	-0.1315***	-0.1282***	-0.1285***
	[0.0371]	[0.0372]	[0.0373]	[0.0373]
Marital Status	0.3195	0.2932	0.3098	0.3091
	[0.3202]	[0.3189]	[0.3190]	[0.3189]
Year of Education	-0.0171	-0.0146	-0.015	-0.0169
	[0.0284]	[0.0284]	[0.0283]	[0.0283]
Asset of Household	1.20E-06	1.41E-06	1.43E-06	1.19E-06
	[1.76e-06]	[1.79e-06]	[1.78e-06]	[1.77e-06]
_cons	2.1372	4.079	3.4554	1.8169
	[13.6481]	[13.4966]	[13.5908]	[13.6100]
N	1298	1298	1298	1298
F	9.7559	8.7237	9.8704	7.8479

Table S6 (Full table of Table 8 in manuscript)

	(1)	(2)	(3)	(4)	(5)
	Time Spent on Domestic Paid Work	Time Spent on Domestic Paid Work	Area of Self-cultivated Farmland	Number of Friends	Number of Close Friends
Agricultural Cooperatives	0.41159*	0.41159**	0.56438*	0.22752**	0.16321*
	(0.21184)	(0.19879)	(0.32053)	(0.09130)	(0.08871)
Sex	0	0	0	0	0
	(.)	(.)	(.)	(.)	(.)
Age	-0.24228	-0.24228	-0.74537	0.39618**	0.36874**
	(0.32078)	(0.32749)	(0.69664)	(0.18954)	(0.18282)
Age_sqr	0.00205	0.00205	0.0053	-0.00184	-0.00122
	(0.00187)	(0.00189)	(0.00358)	(0.00114)	(0.00108)
Sr_health	0.15833	0.15833	0.24941	0.09979*	0.09464*
	(0.11409)	(0.11413)	(0.16514)	(0.05458)	(0.05017)
ADL	-0.0006	-0.0006	0.02314	-0.00013	0.0024
	(0.00983)	(0.01010)	(0.01995)	(0.00640)	(0.00636)
IADL	-0.07764*	-0.07764*	0.01526	-0.04926*	-0.04464*
	(0.04464)	(0.04373)	(0.07388)	(0.02622)	(0.02517)
Marital Status	0.24299	0.24299	0.71052*	-0.06515	0.09491
	(0.37929)	(0.39596)	(0.42029)	(0.20132)	(0.17134)
Year of Education	0.00613	0.00613	0.08374	-0.02434	-0.02294
	(0.05044)	(0.05272)	(0.07223)	(0.02310)	(0.02113)
Asset of Household	0	0	0	0	0
	(0.00000)	(0.00000)	(0.00000)	(0.00000)	(0.00000)
_cons	9.19307	9.76912	26.89319	-17.20163**	-18.95423**
	(14.79788)	(15.48243)	(33.97269)	(8.50019)	(8.24629)
N	1338	1338	1338	1338	1338
F	1.41762		1.50426	2.54693	2.82993
r2	0.0122		0.0142	0.0229	0.0249

Notes: Bootstrap standard error used in Column 2.

Table S7 (Full table of Table 9 in manuscript)

	(1)	(2)	(3)	(4)
	Time Spent on Physical Exercise	Time Spent on Physical Exercise	Time Spent on Social Interaction	Time Spent on Social Interaction
Cultural and Sports Clubs	0.04651	0.04651	0.28608*	0.28608*
	(0.15053)	(0.10338)	(0.14782)	(0.15778)
Sex	0	0	0	0
	(.)	(.)	(.)	(.)
Age	-0.02539	-0.02539	0.43808	0.43808
	(0.24261)	(0.18042)	(0.28778)	(0.29111)
Age_sqr	-0.00016	-0.00016	-0.00299**	-0.00299**
	(0.00132)	(0.00109)	(0.00140)	(0.00139)
Sr_health	-0.02182	-0.02182	0.01205	0.01205
	(0.03989)	(0.04955)	(0.06879)	(0.06684)
ADL	0.01465**	0.01465***	0.01465**	0.01465**
	(0.00519)	(0.00507)	(0.00707)	(0.00679)
IADL	0.02761	0.02761	0.06170**	0.06170*
	(0.01901)	(0.02367)	(0.03131)	(0.03219)
Marital Status	-0.14931	-0.14931	0.05955	0.05955
	(0.24106)	(0.18604)	(0.25234)	(0.25549)
Year of Education	-0.01442	-0.01442	0.04852*	0.04852*
	(0.01950)	(0.01938)	(0.02836)	(0.02786)
Asset of Household	0	0	0	0
	(0.00000)	(0.00000)	(0.00000)	(0.00000)
_cons	2.28525	2.0188	-16.19973	-16.12667
	(10.94480)	(8.05854)	(14.66254)	(14.78183)
N	1338	1338	1338	1338
F	10.39387		2.15355	
r2	0.012		0.0203	

Notes: Bootstrap standard error used in Columns 2 and 4.
